# The Effect of Physical Faults on a Three-Shaft Gas Turbine Performance at Full- and Part-Load Operation

**DOI:** 10.3390/s22197150

**Published:** 2022-09-21

**Authors:** Waleligne Molla Salilew, Zainal Ambri Abdul Karim, Tamiru Alemu Lemma, Amare Desalegn Fentaye, Konstantinos G. Kyprianidis

**Affiliations:** 1 Mechanical Engineering Department, Universiti Teknologi PETRONAS, Bandar Seri Iskandar 32610, Perak, Malaysia; 2 Centre for Automotive Research and Electric Mobility (CAREM), Universiti Teknologi PETRONAS, Bandar Seri Iskandar 32610, Perak, Malaysia; 3 School of Business, Society and Engineering, Mälardalen University, Box 883, SE-72123 Västerås, Sweden

**Keywords:** gas turbine, performance model, steady-state, full-load, part-load performance, physical faults

## Abstract

A gas path analysis approach of dynamic modelling was used to examine the gas turbine performance. This study presents an investigation of the effect of physical faults on the performance of a three-shaft gas turbine at full-load and part-load operation. A nonlinear steady state performance model was developed and validated. The datasheet from the engine manufacturer was used to gather the input and validation data. Some engineering judgement and optimization were used. Following validation of the engine performance model with the engine manufacturer data using physical fault and component health parameter relationships, physical faults were implanted into the performance model to evaluate the performance characteristics of the gas turbine at degradation state at full- and part-load operation. The impact of erosion and fouling on the gas turbine output parameters, component measurement parameters, and the impact of degraded components on another primary component of the engine have been investigated. The simulation results show that the deviation in the output parameters and component isentropic efficiency due to compressor fouling and erosion is linear with the load variation, but it is almost nonlinear for the downstream components. The results are discussed following the plots.

## 1. Introduction

A component of a gas turbine engine may deteriorate over time because of hostile operating conditions. The most challenging task for engine users is to ensure a high level of reliability and availability throughout the entire operational lifetime while maintaining efficiency. Identification of engine component health status, the causes of deterioration, and evaluation of engine performance in terms of economic profit and dependability are highly beneficial regarding gas turbines [1,2]. Common gas path faults are fouling, erosion, corrosion, seal clearance and blade tip, thermal distortion, and domestic and foreign object damage. Among them, erosion and fouling impose the highest impact on the performance degradation of the gas turbine [3,4]. Degradation of gas turbine performance has a substantial impact on a power plant’s economic viability [5]. This would lead to significant energy consumption, decreased productivity, noise, and severe machinery breakage. The performance of a gas turbine may be deteriorated either temporarily or permanently. The first can be recovered in part during engine maintenance and operation, while the second requires complete repair [6]. For example, fouling leads to temporary deterioration, while airfoil distortion leads to permanent deterioration. Permanent degradation is neither recovered by washing nor overhaul [7]. Degradation can still be classified as recoverable (with washing) or non-recoverable (cannot be retrieved with washing during operation but can be retrieved after overhaul). Furthermore, performance degradation is categorized as rapid and gradual degradation as per the engine’s service period or the propagation time frame of the degradation [8]. Rapid deterioration is called short-term degradation and occurs during the early stages of the engine’s operation or because of a specific incident, such as foreign or domestic object damage. Gradually, damage occurs in the long-term because of the intake and deposition of various pollutants, as well as a relatively high temperature. In actuality, the deviation spool speed, pressure, fuel flow, and temperature from a baseline will always provide signs of component degradation. The overall performance of the engine can be affected by changes in load or ambient conditions [9]. Thus, developing the gas turbine performance calculation model based on the gas path analysis approach will assist to accurately judge the operating status of the engine and reasonably arrange maintenance and repair work [5]. This improves the reliability and availability of the gas turbine [10,11]. Thus, the development of engine maintenance techniques has been motivated by the aim of achieving high reliability, availability, and efficiency in gas turbines [12].

Gas path analysis (GPA), first developed by Urban [13], has been demonstrated by Talebi and Tousi [14] to be one of the most reliable technologies for engine health monitoring and is frequently used for gas turbine condition monitoring to detect, identify, and evaluate component degradation. From a financial point of view, the engine’s performance loss is significantly impacted by the deterioration of gas turbine components [15]. The model-based diagnosis method demands deep specialist knowledge on the gas turbine model’s development and is challenging to match the components of the gas turbine engine. The second type of gas turbine diagnosis method is data-driven, which exhibits machine learning or deep learning [16]. Finding new sets of data that were not used during the training phase is the more important task for the data-driven method [17]. A hybrid approach, which is a combination of two or more data-driven approaches, is used to offset one’s technique limitation. Because there is always a trade-off when choosing a diagnostic approach, including accuracy, computational performance, and measurement noise, a model-based approach is recommendable because of its flexibility and noise-free nature [18]. Model-based methods will be the focus of this study since fast decision-making, ensuring efficient, robust, trustworthy, and real-time diagnosis, is essential. The performance of the diagnosis might be severely affected by the number of concurrent fault components. Nonlinear diagnostic systems become much more sophisticated when there are more than two damaged components [19].

Development of a performance model of a gas turbine has two main tasks: design point model development and off-design model development. The design point performance model is the model at a single operating point (maximum operating point), while off-design is development of the performance model at the entire operating point. The off-design performance model relies on a component performance map [20]. Iteration algorithms have been used by scholars to solve the off-design problem [21,22]. The first approach, which is most often used, chooses one or perhaps more iterative variables based on the engine configuration and attempts to satisfy the compatibility equations by altering the values. Iteration loops have been used to accomplish this task [23,24]. Researchers who perform off-design analysis commonly use this approach as they are usually focused on simple gas turbines [25]. This method is easier and more effective than others for simple gas turbine engines, which often have only one iteration variable. However, if the number of engine components and complexity rise, an additional variable will be considered. The second approach is related to the first, although the compatibility equations are solved using the quasi-Newton methods [20], Newton–Raphson iteration technique [26], and minimization process [27]. The selection of iteration variables in the off-design analysis in the approach requires significant experience, knowledge, and understanding of the behavior of the pertinent gas turbine engine [28]. In terms of thermodynamic calculation, developing the performance can also be completed using a variety of tools, including commercial software [29]. Most of those software programs require a commercial license and do not allow additional components to be added to their library. Furthermore, they cannot be integrated with other applications, such as MATLAB, for optimization purposes.

Several studies have been carried out to analyze performance deviation from the clean condition of the engine due to fouling, erosion, inlet temperature variation, and bleed air leakage. Based on a heuristic optimization strategy, Tsoutsanis et al. [30] proposed an adaptive diagnostics method for identifying compressor degradation through map tuning. Increasing the number of deteriorated components, on the other hand, may be effective for local optimal solutions but not for global optimal solutions. Using appropriate axis transformations, Al-Hamdan and Ebaid [31] studied the modelling and simulation of a gas turbine for power generation by superimposing a turbine map on a compressor map. For forecasting the part-load performance of aero-derivative gas turbines, Haglind and Elmegaard [32] suggested two models. In one, the compressor and turbine were represented by actual maps, whereas, in the other, a compressor map with turbine constants was used. Both models were discovered to be capable of making accurate forecasts of mass flow and pressure ratio profiles across 40–100% loads, as well as thermal efficiency and exhaust temperatures for part-loads above 70%. A general simulation program for basic, recuperative, and reheat gas turbines was created by Lee et al. [25]. For the compressor, a stage-stacking approach with a general stage map was utilized, whereas, for the turbine, a stage-by-stage model with efficiency correction was used. The prediction accuracy of the algorithm for part-load performance remained unexplored because it had only been validated under full-load conditions. Using thorough compressor and turbine maps, Song et al. [33] created a performance analysis model for a three-spool gas turbine and showed that the model was accurate in forecasting gas turbine performance. All these models require precise performance maps of the air compressor and turbine to predict gas turbine performance [34]. Real performance maps, in practice, are the exclusive property of engine manufacturers and usually unknown to gas turbine users. Thus, it is necessary to develop models using well-designed commercial software to predict gas turbine performance accurately. In addition to this, in the above studies, VIGV scheduling and bleed air were not considered.

Cyrus et al. [6] developed a performance model and the topic of recoverable and nonrecoverable deterioration of gas turbines was extensively covered. The mechanisms of deterioration, such as compressor and turbine fouling, erosion, increasing clearances, and seal distress, were discussed along with their appearances, best practices, and methods of mitigation. Furthermore, permanent degeneration was addressed. Gas turbine input filtration and compressor washing were treated in detail due to the significance of compressor fouling. Along with simulations of typical deterioration modes of gas turbine combined cycles, approaches for performance monitoring of steady-state and transient behavior were provided. A brief explanation of heat recovery steam generator performance monitoring was presented as the usage of many gas turbines in cogeneration and combined cycles. To investigate the combined effects of fouling and changes in environmental conditions on gas turbine performance, Muhammad et al. [35] developed a single-shaft industrial gas turbine performance model. The case engine was a variable geometry engine, and the research identified that, in hot and humid conditions, a rise in the temperature of the intake air causes a reduction in engine performance. Finally, the researchers concluded that integrating inlet air cooling can reduce the engine performance deterioration caused by ambient condition variation, particularly in hot climate zones. For industrial twin-shaft gas turbines that have variable geometry in the compressors and turbines, in addition to control of fuel running at various ambient temperatures, Sepehr et al. [36] created and discussed an innovative approach. The research concentrated on off-design with fluctuating environmental temperatures. They developed a gas turbine performance model by employing compressor and turbine characteristic maps and using the thermodynamic matching method and genetic algorithm optimization strategy. The power output was as an objective function.

In order to corelate the measured data and track how fault occurrence impacts the model parameters, Merrington et al. [37] created a mathematical performance model. The results obtained show a notable improvement over those attained using traditional procedures. A sequential-model-based method for analyzing the performance of the gas turbine was created by Yu-Zhi Chen et al. [38]. In comparison to past model-based methods that required the same number of measurements, the new method is effective because it can more precisely and successfully assess the level of engine component degradation. The conclusion was reached that the new approach offers an accurate diagnostic with a smaller set of measurements. The most effective gas turbine diagnosis model was created by Diakunchak [39]. His research categorized gas turbine performance degradation into two categories: recoverable deterioration and unrecoverable deterioration. Additionally, he investigated the main reasons for performance decrease and drop in performance indicators. Moreover, he suggested strategies for monitoring and predicting the deterioration in the performance of the gas turbine. Escher [40] developed a model for gas turbine performance and provided suggestions about how to investigate common gas-path problems and how they relate to important performance indicators. He also developed the PYTHIA software to develop the gas turbine performance decline model using Newton–Raphson solution techniques. The simulation results were also used in the construction of the gas-path fault diagnosis system.

MacLeod et al. [41] built a performance model and studied the performance effects of common physical faults on each individual engine component. A mathematical model for a gas turbine was created by Aker et al. [42] to investigate the effect of fouling in compressor blades and to capture gas-path fault indicators for fault identification and to lower excessive unscheduled maintenance costs and overhead costs. They also examined how compressor fouling affected gas turbine key performance parameters. Saravanamuttoo and Maclsaac [43] developed an engine performance model to study the correlation of measurement and health parameters. The performance degradation model, which was essentially used to analyze the engine performance deterioration, was provided. It was based on the conventional gas turbine model, and a modified deterioration component characteristic curve was used to represent gas turbine gas-path faults. Lakshminarasimha et al. [44] built models of gas turbines and examined how erosion causes degradation in an aircraft engine and fouling in a power plant engine.

**Figure 1:**
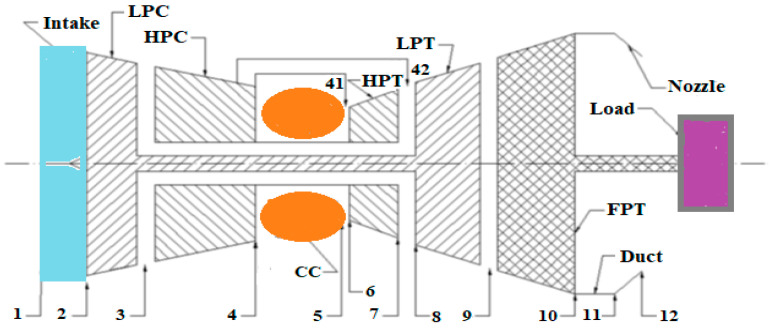
Three-shaft gas turbine configuration.

Although researchers have worked on gas turbine engine performance modeling, the part-load performance of the RB211-24G gas turbine has remained unexplored. In addition to this, the open literature review reveals that a secondary air system and a variable inlet guide vane (VIGV) schedule have not been considered. A variable inlet guide vane is mounted to the low-pressure compressor. It is used to monitor the inlet flow rate and to prevent the gas turbine from surging. The VIGV nominal angle is always scheduled as a function of non-dimensional low-pressure compressor spool speed. Through various linkages attached to guide vanes, an actuation mechanism operates the VIGVs. When the non-dimensional low-pressure compressor speed lowers because of fouling or erosion, the variable inlet guide vane is opened more to increase the air mass flow rate and maintain efficiency. It is also used to maintain the gas turbine efficiency at the start-up and shut-down periods of the engine. Thus, this study aims to investigate the impact of physical faults on gas turbine engine performance at full- and part-load operation while considering VIGV scheduling and a secondary air system. Since erosion and fouling are the common problems in gas turbines, the focus of this research is on the effect of these two faults on the gas turbine performance at part-load operation. The following structure is followed to present this paper. First, a brief overview is presented of the essential components of a gas turbine and how their performance degrades, as well as its behavior. Second, an examination takes place regarding how the three-shaft nonlinear steady-state model for gas turbines was built. Third, the types of physical faults and simulation of gas-path faults on main components are discussed. Finally, a discussion takes place regarding the simulated part-load performance. To aid the development of the gas turbine diagnosis model, the main gas-path faults indicator measurement parameters are examined and discussed. GasTurb 13 created by Jaochim Kurzke, Aachen, Germany, commercial software was purchased and used to develop both the design point performance model and off-design performance model. The design point performance model and off-design performance model input data are collected from the engine datasheet. The variable inlet guide vane schedule and the off-design performance model validation data are also collected from the engine datasheet. The case engine is a three-shaft industrial gas turbine engine Manufactured by Rolls Royce, Goodwood, England. The engine model number is RB211-24G. LPC, HPC CC, HPT, LPT, and PT are the six primary gas-path components [45]. Figure 1 depicts a three-shaft gas turbine configuration.

## 2. The Development of a Gas Turbine Engine’s Performance Model

**Figure 2:**
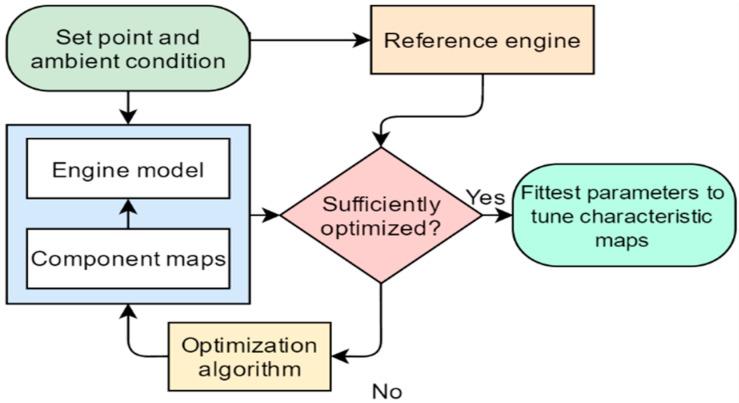
Development of engine performance model via component map tuning.

The performance characteristics of a gas turbine engine’s individual components and the compatibility principles for work, mass flow, efficiency, and rotational speed, which govern how components are matched, determine the engine’s overall performance [46]. Two efforts are required to model the performance of gas turbines: development of design point and off-design [35]. The overall performance is determined by integrating the performance of all such components [47]. Reducing experimental data to a minimum and calculating unknown parameters using the performance model is a cost-effective solution to this problem [48]. The process of developing engine performance via component map tuning is depicted in Figure 2. The data for the design point input were gathered from the manufacturer’s technical datasheet. An energy-based approach was used during development of the performance model [49]. If the model results closely match those obtained from OEM data, the model is considered as an accurate model [50]. Otherwise, the model still needs to be optimized.

### 2.1. Design Point Performance Model

Design point calculations were conducted to ascertain the parameters for the engine’s maximum power output. Some of the input data were not available in the engine datasheet, which is because of proprietary issues: engine manufacturers are not sharing all the necessary data with end-users. To address this problem, using thermodynamic and compatibility equations, design point performance evaluation will determine the unknown design parameters. The model was created with the use of data from the catalogue, open literature, and some empirical approximations and engineering assumptions. The product catalogue lists the power rating and spool speeds. The ambient condition used to determine the design point performance model was 288.15K, which is the ISO standard temperature. The gas turbine’s overall single cycle thermodynamic performance model is the combination of separate component models. Three-shaft gas turbine engines were the focus of this study. The inlet duct, low-pressure compressor (LPC), high-pressure compressor (HPC), combustion chamber (CC), high-pressure turbine (HPT), low-pressure turbine (LPT), power turbine (PT), and exhaust duct are the main components of the engine. All components require detail modelling. The design point calculation’s major goal was to acquire all data at a specific operating point. Almost all main design parameters were collected in the manufacturer technical documents. However, some gas path measurements and performance parameters, such as P2, T2, T3, P4, T3, P4, T5, P5, T6, P6, P7, wf, and each component efficiency, were among the unavailable parameters that needed to be determined. The input data shown in Table 1 were used to develop the design point model and to simulate all the required parameters. Table 2, Table 3 and Table 4 show the optimization parameters, constraints, and objective function that were used to optimize the model.

Finally, it was discovered that the design point model output closely matched with the engine OEM’s data, with just minor differences. The design point calculation result was compared to design parameters found in the gas turbine manufacturer datasheet. As indicated in Table 5, the % error in each parameter variation from the engine datasheet was quite low. As a conclusion, it was discovered that the design point values produced by the GasTurb 13 simulation are quite reliable as they closely matched with the actual design point values.

### 2.2. Off-Design Performance Model

After successful matching of the design point performance model, an off-design model was created and validated. Off-design performance refers to a gas turbine’s capability to run for extended periods of time under conditions different than the first cycle point. An off-design state can be caused by changes in ambient conditions and engine load. For example, the ambient temperature might greatly differ from summer to winter, which affects engine performance substantially. Hence, developing a design model requires that the engine will work well in both design and off-design settings. The first task of off-design simulations is to match the design point values to the defined compressor and turbine maps using different scaling techniques. In this research, the appropriate compressor and turbine maps were selected and scaled to the design point values. Component matching is the second step of off-design, which is completed using the Newton–Raphson iterative technique to ensure mass flow and work compatibility [35,51]. An auxiliary coordinate called beta was added during the component map digitization process, as suggested by Kurzke [45], to eliminate discrepancy. Component maps show the relationship between the isentropic efficiency, pressure ratio, corrected mass flow rate, and rotational speed. The rotational speed (rpm), intake mass flow rate (kg/s), inlet temperature (K), and inlet pressure to the compressor or turbine are the primary parameters in the compressor and turbine map (kPa). Standardizing corrected mass flow, corrected speed, isentropic efficiency, and pressure ratio with design point values makes using performance maps easier.

To make it comparable with the cycle design point efficiency dp and corrected flow, the value generated from the map table, such as efficiency, pressure ratio, corrected mass flow, and corrected speed, must be corrected for Reynolds number effects, which is called Reynolds number index (*RNI*), with the terms *f_η_,_RNI_* and *f_W_,_RNI_*. The map scaling point in GasTurb is set at *ß_R_,_map_* = 0.5 and *N_R_,_map_* = 1.0 by default. The map scaling point (subscript *_R_,_map_*) is used as a reference point where the design point (subscript *dp*) is matched.

(1) $$\eta_{dp,map\;}=\eta_{R,map}\cdot f_{\eta ,RNI}$$

(2) $${\left(\frac{W\sqrt{\Theta_{R}}}{\delta }\right)}_{dp,\;map}\;={\left(\frac{W\sqrt{\Theta_{R}}}{\delta }\right)}_{R,\;map\;}\cdot f_{W,RNI}$$

where *f_η_,_RNI_* is the Reynolds number index to correct the reference point efficiency read from the map, *η_dp,map_* is the scaled map design point efficiency, ${\left(W\sqrt{\Theta_{R}})/\delta \right)}_{\mathrm{dp},\mathrm{map}}$ is the scaled map corrected mass flow, *η_R_,_map_* is the reference point efficiency from unscaled map, and ${\left(W\sqrt{\Theta_{R}})/\delta \right)}_{\mathrm{dp},\mathrm{map}}$ the reference point corrected mass flow from unscaled map. The corrected temperature (Θ) and corrected pressure (*δ*) are expressed below; *f_W_,_RNI_* is the Reynolds number index to correct the reference point corrected mass flow read from the map.

$$\theta =\frac{T_{0}}{288.15K^{\prime }}$$

(3) $$\delta =\frac{P_{0}}{101.325\mathrm{KPa}}$$

where *T*_0_ and *P*_0_ are the inlet temperature and pressure. The following scaling factors were determined by assuming *f_η__,RNI_* = 0.99 and *f_η__,RNI_* = 0.995 [29].

(4) $$f_{\mathrm{Mass}\;}=\frac{{\left(\frac{W\sqrt{\Theta_{R}}}{\delta }\right)}_{dp}}{{\left(\frac{W\sqrt{\Theta_{R}}}{\delta }\right)}_{R,\mathrm{map}\;}\cdot f_{W,RNI}}$$

(5) $$f_{Eff}=\frac{\eta_{dp}}{\eta_{R,\mathrm{map}\;}\cdot f_{\eta ,RNI}}$$

(6) $$f_{P_{3}/P_{2}}=\frac{{\left(P_{3}/P_{2}\right)}_{dp}-1}{{\left(\frac{P_{3}}{P_{2}}\right)}_{R,\mathrm{map}\;}-1}$$

(7) $$f_{\mathrm{Speed}\;}=\frac{1}{N_{R,\mathrm{map}\;}}$$

where *f_Mass,_* *f_Eff,_* *f*_*p*_2_*/p*_3__, and *f_Speed_* are mass flowrate, efficiency, pressure ratio, and speed scaling factors, respectively. *P*_2_ and *P*_3_ are compressor inlet and exit inlet pressure. Additionally, *N_R_,_map_* is compressor speed in the map. Once all these scaling factors have been established, efficiency, corrected mass flow, corrected speed, and pressure ratio must be multiplied by the scaling factors in order to scale the map. Table 6, Table 7 and Table 8 show the spool speed, surge margin, and map coordinates, respectively, which are of the scaled maps information.

**Figure 3:**
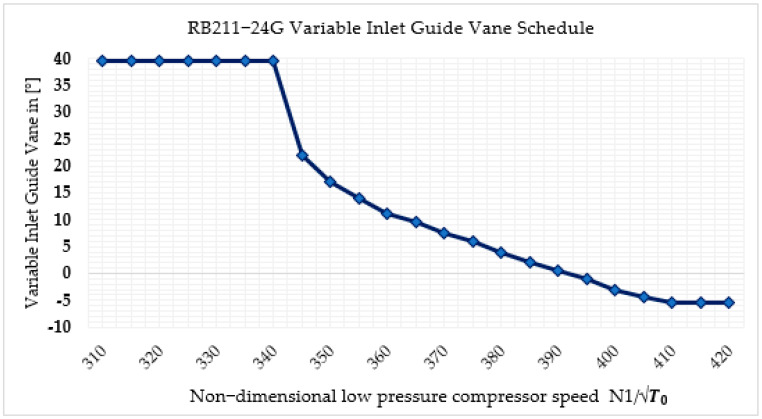
VIGV schedule.

The necessary component maps were chosen, and the design point was correlated for realistic off-design simulations. The off-design model was developed and confirmed with the OEM data after the map was scaled to the design point. Catalog data, such as power output versus ambient temperature and efficiency versus ambient temperature, were used to validate the off-design model. The variable inlet guide vane scheduling has a significant contribution to the gas turbine’s performance. It helps to maintain the thermal efficiency of the engine by controlling the mass flow rate. In the RB211-24G-RT56, the VIGV has 18 blades and is mounted in the LPC in the actual engine. The VIGV is always placed at a nominal angle with respect to non-dimensional low-pressure compressor spool speed. The VIGV schedule for RB211-24G-RT56, which is collected from the original engine manufacturer, is shown in Figure 3. In this study, during simulation, both VIGV scheduling and secondary air systems were taken into account. GasTurb 13 commercial software has an option to consider VIGV. To incorporate the schedule into the model, first of all, under the “Var Geometry” section, the VIGV has to be activated by selecting the low-pressure compressor where the variable inlet guide vane is to be mounted. This can be completed by manipulating three numbers, such as 0, 2, and 3. Zero indicates that there is no VIGV in the engine; it means the engine is at a fixed position throughout the operation. Number two indicates that the engine has VIGV and is mounted to a low-pressure compressor. Number three indicates that the engine has VIGV and is mounted to a high-pressure compressor. Since the case engine, RB211-24G-RT56, was originally designed with VIGV and to be mounted to the low-pressure compressor, number two was selected and VIGV was activated. Once the VIGV is activated, the next step is incorporating the VIGV schedule graph using the “Controls” and then “Schedules” option in GasTurb 13. This option allows one parameter to be a function of another parameter. However, it needs to convert the graph to digital data. After putting the digital data in table form in the software, the software by itself will generate the graph to visualize the schedule. Right after that, the activation in the schedule section, which is indicated by a bulb, should be on to keep the schedule active throughout the simulation.

**Figure 4:**
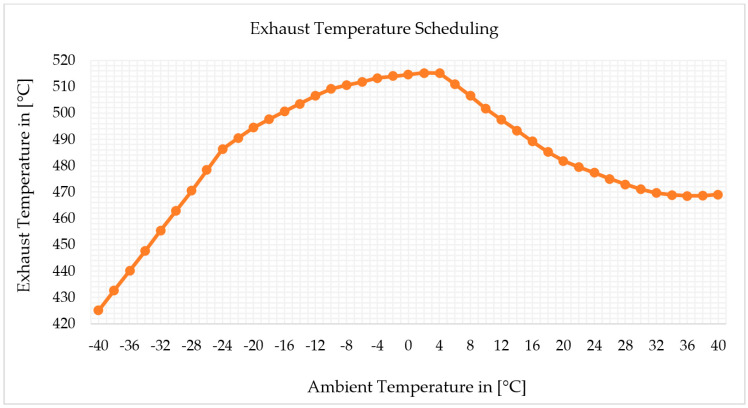
Exhaust temperature scheduling in the simulation.

**Figure 5:**
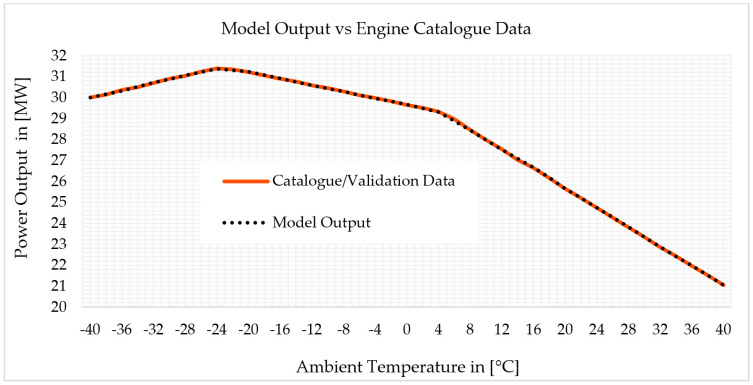
Validation of off-design model with power output (validation data).

Power output versus ambient temperature was iterated over exhaust temperature versus ambient temperature. Figure 4 depicts the exhaust temperature versus ambient temperature schedule incorporated in the software through titration to match the off-design model output to the catalogue data and make the model accurate. The off-design model provides the power output versus ambient temperature after extensive iteration, which was correctly matched with the validation data, as shown in Figure 5. Finally, the output of the model was compared to validation data. At each operating point, the highest variation in power output versus ambient temperature was 0.02%. Figure 5 shows the comparison of the model power output versus ambient temperature with the catalogue data.

The off-design demonstrates a result that has clearly matched with the validation information as shown in Figure 5. It indicates that the model is capable of accurately predicting the gas turbine’s performance under all operating conditions. The model was also simulated and validated with validation data by scheduling the validation data directly, power output versus ambient temperature, into GasTurb 13. In both approaches, the model accuracy is similar, only 0.02% variation. However, scheduling of power output versus ambient temperature into GasTurb 13 has the problem of being unable to simulate part-load operation when the program is already scheduled with the power output remaining constant. Thus, to maintain the power unique at any operating curve, the validation data, power output versus inlet temperature, were scheduled in the software directly to simulate physical faults at full-load, whereas exhaust temperature versus ambient temperature scheduling was used to simulate physical faults at part-load. It is worth noting that the model with exhaust temperature versus ambient temperature scheduling was validated with validation data. The model output with directly scheduling the power output versus ambient temperature graph was identical to the result of iterating the exhaust temperature over power output versus ambient temperature. The main concern is that the power output should be unique throughout the operating curve of the different curves. This means, for example, if the power is fixed at a single point/curve, to the extent that physical faults exist and some parameters vary, the power will be unique compared to other parameters, such as fuel rate and exhaust temperature will be varied to maintain the power. For example, to simulate fouling in compressors, the compressor flow capacity will be reduced by −7.5% and the isentropic efficiency reduction will be −2.5%. Because the output power should be maintained unique after the physical faults have been implanted, the gas generator must create extra high-temperature gas to compensate for the power loss caused by the reduction in component performance parameters. Thus, the flow capacity reduction by −7.5% and isentropic efficiency drop of −2.5% must be checked to the decreased output of the two health parameters after the new power balance point. In short, the change in component performance is divided into two parts: one is due to the fault, as indicated by the drop in flow capacity of 7.5% and isentropic efficiency of −2.5%; the other is due to the imbalance in gas turbine power and some changes in energy matching between components that share a common shaft.

Compressor fouling has been simulated with the intervals of 0–7.5% in mass flow variation and 0–2.5% in isentropic efficiency variation, and the variation relationship between the two parameters is 3:1 [45]. As a result, each 0.25% reduction in efficiency and 0.75% increase in mass flow in the low-pressure compressor and high-pressure compressor fouling is equal to a 10% rise in fault severity. For the other faults studied in this study, each 0.2% reduction in efficiency and 0.4% increase in mass flow correlates to a 10% increase in fault severity. The relationship between physical fault and health measures parameters is depicted in Table 9 [3,45].

## 3. Results and Discussion

### Effect of Fouling and Erosion on the Gas Turbine Output Parameters

Fouling and erosion were simulated using the relationship between physical faults and health factors shown in Table 9. Physical faults were injected using GasTurb 13’s Modifier option. The independent variables are isentropic efficiency and flow capacity, and changes in these independent factors cause changes in component performance. Physical faults in the gas turbine are quantified using these performance parameters. As a result of the change in the independent parameters, the dependent parameters, such as pressure, temperature, fuel flow, and power output, vary. Changes in the compressor isentropic efficiency and flow capacity (independent parameters) were analyzed to quantify the fouling and erosion phenomena in the compressor and turbine. When simulating the degradation model, component characteristics maps must be updated to account for deterioration. The maps stored in GasTurb 13 are for a clean engine. As a result, the compressor map’s scaling factors must be modified in response to changes in the independent parameters for a deteriorating engine. The scaling factors for mass flow, isentropic efficiency, and pressure ratio will be determined as shown below [41].

(8) $$SF_{\Gamma ,C}=1+\frac{\Delta H_{\Gamma ,C}}{100}\;$$

(9) $$SF_{\Gamma ,C}=1+\frac{\Delta H_{\eta ,C}}{100}\;$$

(10) $$SF_{\Gamma ,T}=1+\frac{\Delta H_{\Gamma ,T}}{100}$$

(11) $$SF_{\eta ,T}=1+\frac{\Delta H_{\eta ,T}}{100}$$

where $SF_{\Gamma ,C}$ and $SF_{\Gamma ,C}$ describe the scaling factors used to scale the compressor flow capacity and efficiency, $SF_{\Gamma ,T}\;\mathrm{and}\;SF_{\eta ,T}$ describe the scaling factors used to scale the turbine compressor flow capacity and efficiency, $\Delta H_{\Gamma ,T}$ and $\Delta H_{\eta ,T}$ represent variation amounts of turbine health parameters, while $\Delta H_{\Gamma ,C}$ and $\Delta H_{\eta ,C}$ are for variation quantities of compressor health parameters.

The effect of erosion and fouling severity on exhaust temperature (T5), specific fuel consumption (SFC), thermal efficiency (ηTH), pressure ratio (PR), turbine inlet temperature (TIT), and heat rate (HR) were simulated after physical faults were implanted with application of the relations mentioned above in Table 9. Fouling and erosion severity are applied to each of the five components.

**Figure 6:**
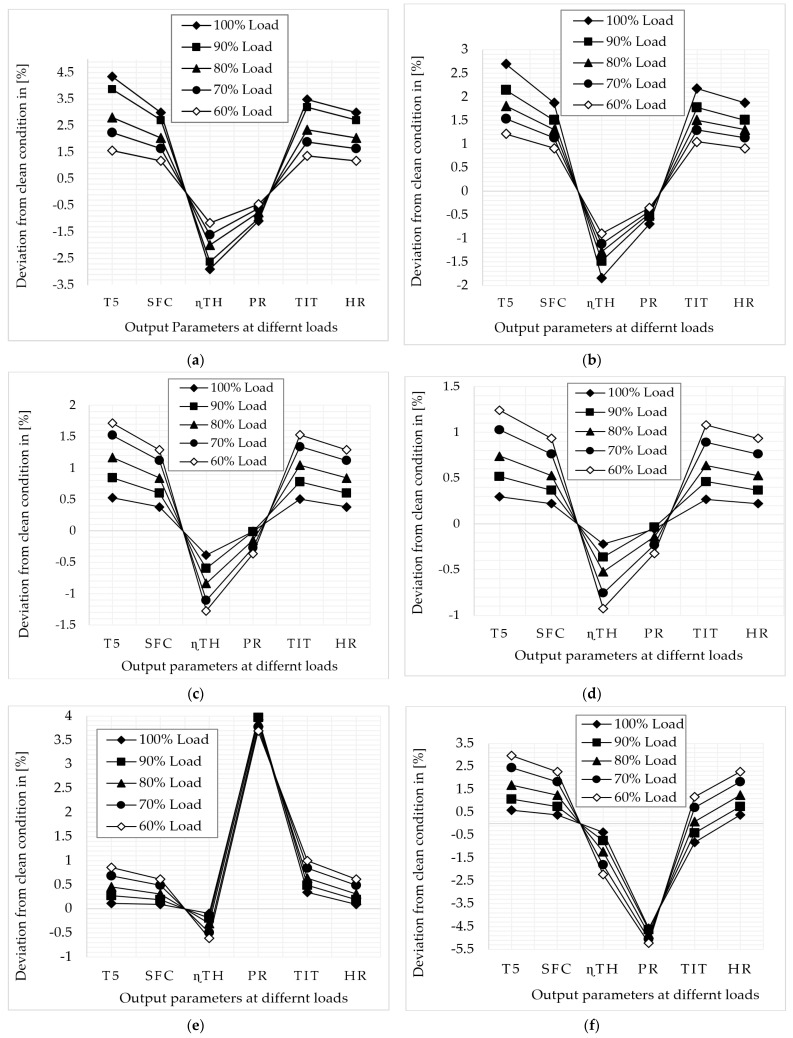

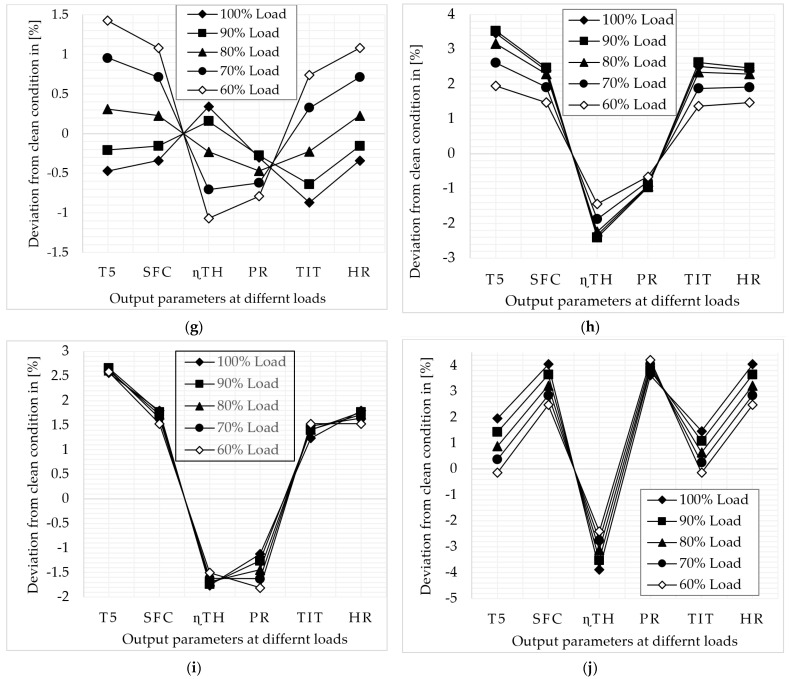
Deviation in output parameters due to physical faults at full−load and part−load operation: (**a**) LPC fouling at 100% fault severity, (**b**) LPC erosion at 100% fault severity, (**c**) HPC fouling at 100% fault severity, (**d**) HPC erosion at 100% fault severity, (**e**) HPT fouling at 100% fault severity, (**f**) HPT erosion at 100% fault severity, (**g**) LPT fouling at 100% fault severity, (**h**) LPT erosion at 100% fault severity, (**i**) PT fouling at 100% fault severity, (**j**) PT erosion at 100% fault severity.

The above graphs clearly show how fouling and erosion affect gas turbine output parameters at full-load and part-load operation. Figure 6a shows the LPC fouling effect, and the simulation result shows that the maximum deviation that occurs in exhaust temperature is about 3.86% at 90% load, 2.8% at 80% load, 2.22% at 70% load, and 1.55% at 60% load, whereas the minimum deviation that occurs in pressure ratio is about −0.99% at 90% load, −0.76% at 80% load, −0.61% at 70% load, and −0.46% at 60% load. The deviation from the clean condition rises as the load increases. The exhaust temperature, specific fuel consumption, turbine inlet temperature, and heat rate are increasing, whereas thermal efficiency and pressure ratio are decreasing. Figure 6b shows the LPC erosion effect, and the simulation result shows that the maximum deviation that occurs in exhaust temperature is about 2.14% at 90% load, 1.8% at 80% load, 1.5% at 70% load, and 1.2% at 60% load, whereas the minimum that deviation occurs in pressure ratio is about −0.53% at 90% load, −0.48% at 80% load, 0.42% at 70% load, and 0.36% at 60% load. The deviation from the clean condition rises as the load increases. The exhaust temperature, specific fuel consumption, turbine inlet temperature, and heat rate are increasing, whereas thermal efficiency and pressure ratio are decreasing.

Figure 6c shows the HPC fouling effect, and the simulation result shows that the maximum deviation that occurs in exhaust temperature is about 0.84% at 90% load, 1.17% at 80% load, 1.52% at 70% load, and 1.71% at 60% load, whereas the minimum deviation that occurs in pressure ratio is about −0.006% at 100% load, −0.011% at 90% load, −0.14% at 80% load, −0.26% at 70% load, and −0.36% at 60% load. The deviation from the clean condition rises as the load decreases. The exhaust temperature, specific fuel consumption, turbine inlet temperature, and heat rate are increasing, whereas thermal efficiency and pressure ratio are decreasing. Figure 6d shows the HPC erosion effect, and the simulation result shows that the maximum deviation that occurs in exhaust temperature is about 0.51% at 90% load, 0.73% at 80% load, 1.02% at 70% load, and 1.24% at 60% load, whereas the minimum deviation that occurs in pressure ratio is about −0.036% at 90% load, −0.14% at 80% load, −0.23% at 70% load, and −0.32% at 60% load. The deviation from the clean condition rises as the load increases. Turbine inlet temperature, specific fuel consumption, exhaust temperature, and heat rate values are increasing, whereas thermal efficiency and pressure ratio are decreasing.

Figure 6e shows the HPT fouling effect, and the simulation result shows that the maximum deviation that occurs in pressure ratio is about 3.96% at 90% load, 3.86% at 80% load, 3.77% at 70% load, and 3.69% at 60% load, whereas the minimum deviation that occurs in thermal efficiency is about −0.19% at 90% load, −0.30% at 80% load, −0.48% at 70% load, and −0.61% at 60% load. The deviation in pressure ratio from the clean condition is decreasing as the load decreases. However, the deviation in thermal efficiency from the clean condition is increasing as the load decreases. The exhaust temperature, specific fuel consumption, turbine inlet temperature, heat rate, and pressure ratio are increasing, whereas thermal efficiency is decreasing. Figure 6f shows the HPT erosion effect, and the simulation result shows that the maximum deviation that occurs in pressure ratio is about −4.63% at 90% load, 4.83% at 80% load, −5.04% at 70% load, and −5.22% at 60% load, whereas the minimum deviation that occurs in thermal efficiency is about −0.74% at 90% load, −1.23% at 80% load, −1.80% at 70% load, and −2.22% at 60% load. The deviation from the clean condition rises as the load increases. The exhaust temperature, specific fuel consumption, and heat rate are increasing, whereas thermal efficiency, turbine inlet temperature, and pressure ratio are decreasing.

Figure 6g shows the LPT fouling effect, and the simulation result shows that the maximum deviation occurs in turbine inlet temperature at 90%; it is about −0.64%, and, at 80%, 70%, and 60% loads, the maximum deviation shown in exhaust temperature is about 0.3% at 80% load, 0.95% at 70% load, and 1.42% at 60% load, whereas the minimum deviation that occurs in pressure ratio at 90% is about −0.27%, and, at 80%, 70%, and 60% loads, the maximum deviation shown in turbine inlet temperature is about −0.22% at 80% load, −0.32% at 70% load, and −0.74% at 60% load. The deviation in turbine inlet temperature from the clean condition at 80%, 70%, and 60% is increasing as the load decreases. Figure 6h shows the LPT erosion effect, and the simulation result shows that the maximum deviation that occurs in exhaust temperature is about 3.53% at 90% load, 3.15% at 80% load, 2.60% at 70% load, and 1.94% at 60% load, whereas the minimum deviation that occurs in pressure ratio is about −0.96% at 90% load, −0.92% at 80% load, −0.80% at 70% load, and −0.65% at 60% load. The deviation in exhaust temperature from the clean condition decreases as the load decreases. The deviation in pressure ratio from the clean condition decreases as the load decreases except at 90% load. The exhaust temperature, specific fuel consumption, turbine inlet temperature, and heat rate are increasing, whereas thermal efficiency and pressure ratio are decreasing.

Figure 6i shows the PT fouling effect, and the simulation result shows that the maximum deviation that occurs in exhaust temperature is about 2.66% at 90% load, 2.61% at 80% load, 2.64% at 70% load, and 2.58% at 60% load, whereas the minimum deviation that occurs in pressure ratio is about −1.25% at 90% load, 1.44 at 80% load, −1.62% at 70% load, and −1.80% at 60% load. The exhaust temperature, specific fuel consumption, turbine inlet temperature, and heat rate are increasing, whereas thermal efficiency and pressure ratio are decreasing. Figure 6j shows the PT erosion effect, and the simulation result shows that the maximum deviation that occurs in specific fuel consumption and heat rate is about 3.64% at 90% load, 3.21% at 80% load, 2.84% at 70% load, and 2.47% at 60% load, whereas the minimum deviation that occurs in turbine inlet temperature is about 1.07% at 90% load, 0.63% at 80% load, 0.24% at 70% load, and −0.14% at 60% load. The exhaust temperature, specific fuel consumption, turbine inlet temperature, pressure ratio, and heat rate are increasing, whereas thermal efficiency is decreasing. The effect of physical faults on the component isentropic efficiencies are shown in the following graphs.

**Figure 7:**
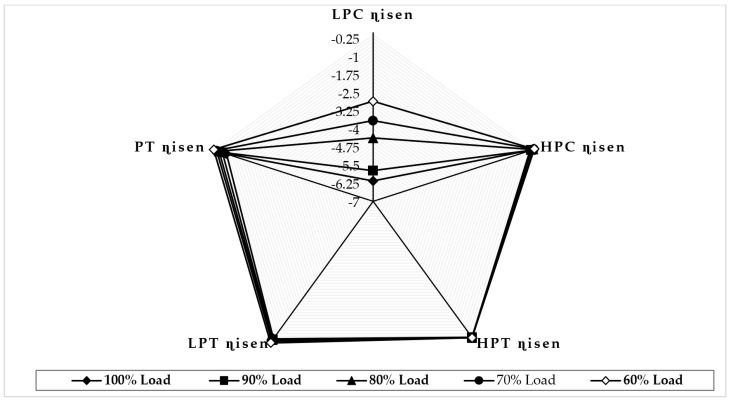
Deviation in component isentropic efficiency due to LPC fouling at 100% fault severity at full−load and part−load operation.

**Figure 8:**
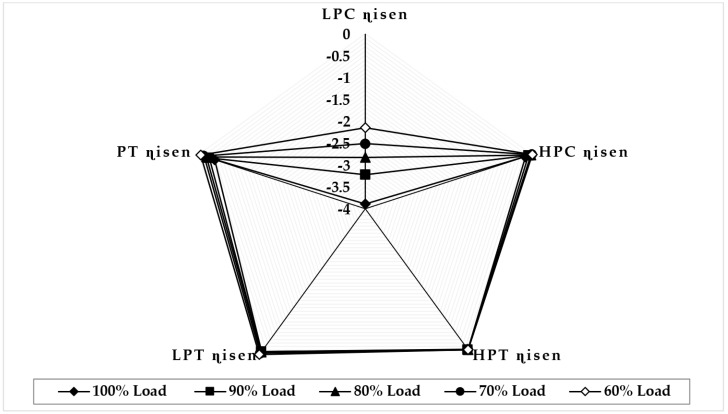
Deviation in component isentropic efficiency due to LPC erosion at 100% fault severity at full−load and part−load operation.

Figure 7 illustrates the variation in component isentropic efficiency when fouling at 100% severity level occurs in the LPC at full-load and part-load operations. The graph depicts that the maximum decrease in isentropic efficiency that occurs on itself is about −5.72 at 90% load, −4.37 at 80% load, −3.65 at 70% load, and −2.84 at 60% load, whereas the minimum decrease in isentropic efficiency shown in HPC isentropic efficiency is about −0.11 at 90% load, −0.026 at 80% load, 0.011 at 70% load, and 0.021 at 60% load. The trend of the deviation in LPC isentropic efficiency is decreasing as loads are decreasing, whereas the HPC isentropic deviation increases as the load decreases. Figure 8 illustrates the variation in component isentropic efficiency when erosion at 100% severity level occurs in the LPC at full-load and part-load operations. The graph depicts that the maximum decrease in isentropic efficiency that occurs on itself is about −3.22 at 90% load, −2.82 at 80% load, −2.51 at 70% load, and −2.14 at 60% load, whereas the minimum decrease in isentropic efficiency shown in HPC isentropic efficiency is about −0.0841 at 90% load, −0.023 at 80% load, 0.0027 at 70% load, and 0.011 at 60% load. The trend of the deviation in LPC isentropic efficiency is decreasing as loads are decreasing, whereas the high-pressure compressor isentropic deviation decreases as the load decreases.

**Figure 9:**
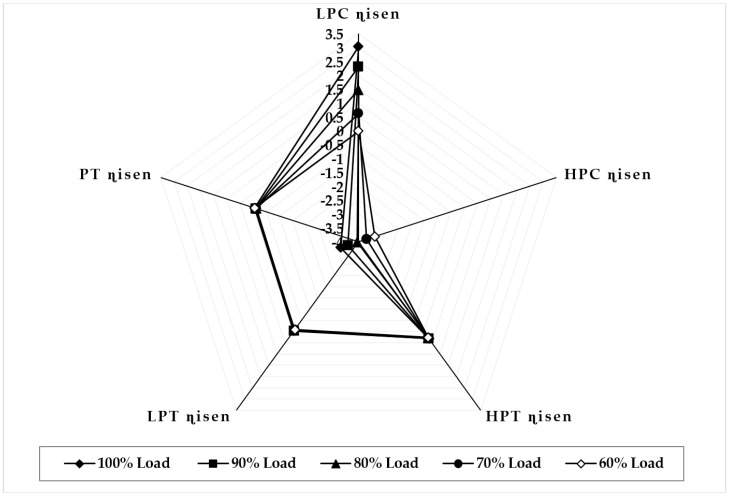
Deviation in component isentropic efficiency due to HPC fouling at 100% fault severity at full−load and part−load operation.

**Figure 10:**
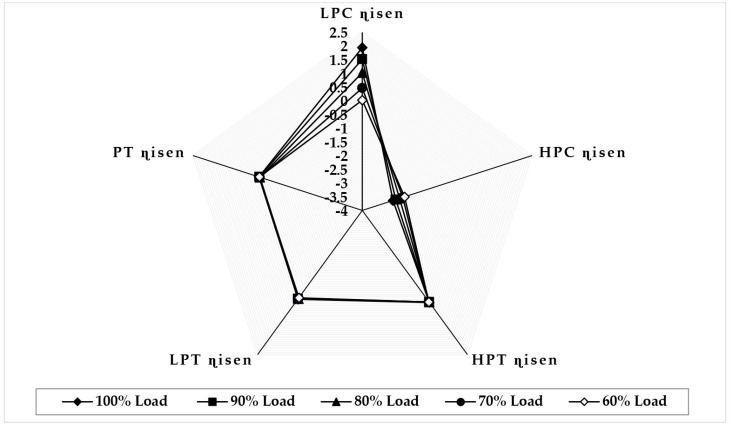
Deviation in component isentropic efficiency due to HPC erosion at 100% fault severity at full−load and part−load operation.

Figure 9 illustrates the variation in component isentropic efficiency when fouling at 100% severity level occurs in the HPC at full-load and part-load operations. The graph depicts that the maximum decrease in isentropic efficiency that occurs on itself is about −4.39 at 90% load, −4.04 at 80% load, −3.68 at 70% load, and −2.14 at 60% load, whereas the minimum decrease in isentropic efficiency observed in LPT isentropic efficiency is about −0.023 at 90% load, −0.047 at 80% load, −0.069 at 70% load, and −0.089 at 60% load. The trend of the deviation in the HPC isentropic efficiency is decreasing as loads are decreasing, whereas the LPC isentropic deviation increases as the load decreases. Figure 10 illustrates the variation in component isentropic efficiency when erosion at 100% severity level occurs in the HPC at full-load and part-load operations. The graph depicts that the maximum decrease in isentropic efficiency that occurs on itself is 1.51 at 90% load, 1.03 at 80% load, 0.46 at 70% load, and 0.023 at 60% load, whereas the minimum decrease in isentropic efficiency shown in LPC isentropic efficiency is about −0.018 at 90% load, −0.028 at 80% load, −0.046 at 70% load, and −0.064 at 60% load. The trend of the deviation in the HPC isentropic efficiency is decreasing as loads are decreasing, whereas the LPT isentropic deviation increases as the load decreases.

**Figure 11:**
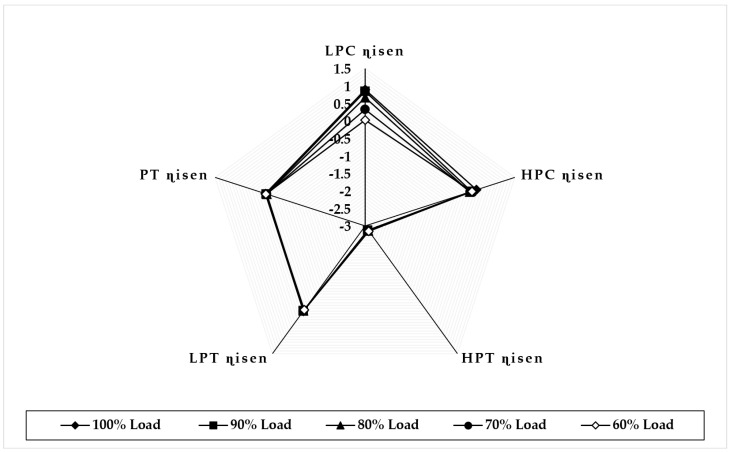
Deviation in component isentropic efficiency due to HPT fouling at 100% fault severity at full−load and part−load operation.

**Figure 12:**
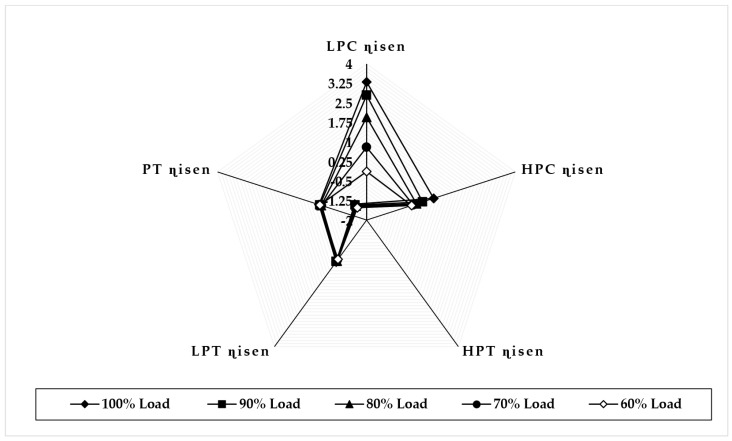
Deviation in component isentropic efficiency due to HPT erosion at 100% fault severity at full−load and part−load operation.

Figure 11 illustrates the variation in component isentropic efficiency when fouling at 100% severity level occurs in the HPT at full-load and part-load operations. The graph depicts that the maximum decrease in isentropic efficiency that occurs on itself is about −2.85 at 100% load, −2.85 at 90% load, −2.83 at 80% load, −2.82 at 70% load, and −2.81 at 60% load, whereas the minimum decrease in isentropic efficiency observed in LPT isentropic efficiency is about −0.009 at 90% load, −0.017 at 80% load, −0.030 at 70% load, and −0.04 at 60% load. The trends of the deviation in both HPT and LPT isentropic efficiencies are decreasing as loads are decreasing. Figure 12 illustrates the variation in component isentropic efficiency when erosion at 100% severity level occurs in the HPT at full-load and part-load operations. The graph depicts that the maximum decrease in isentropic efficiency that occurs on itself is −2.72 at 90% load, −2.68 at 80% load, −2.64 at 70% load, and −2.60 at 60% load, whereas the minimum decrease in isentropic efficiency shown in LPT isentropic efficiency is about 0.025 at 90% load, −0.07 at 80% load, −0.11 at 70% load, and −0.15 at 60% load. The trend of the deviation in the HPC isentropic efficiency is decreasing as loads are decreasing, whereas the LPT isentropic deviation increases as the load decreases.

**Figure 13:**
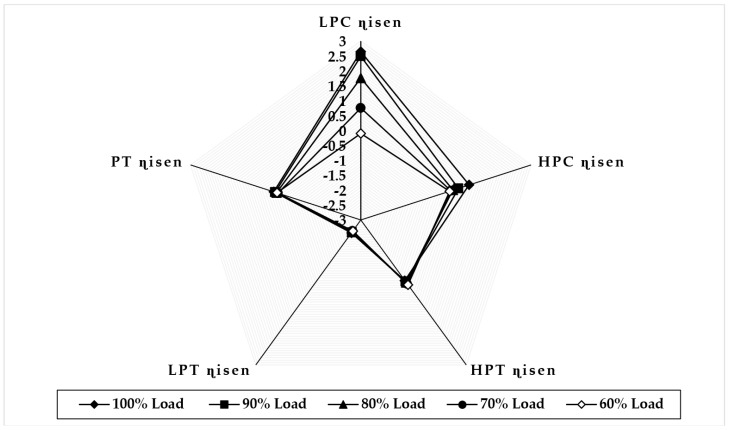
Deviation in component isentropic efficiency due to LPT fouling at 100% fault severity at full−load and part−load operation.

**Figure 14:**
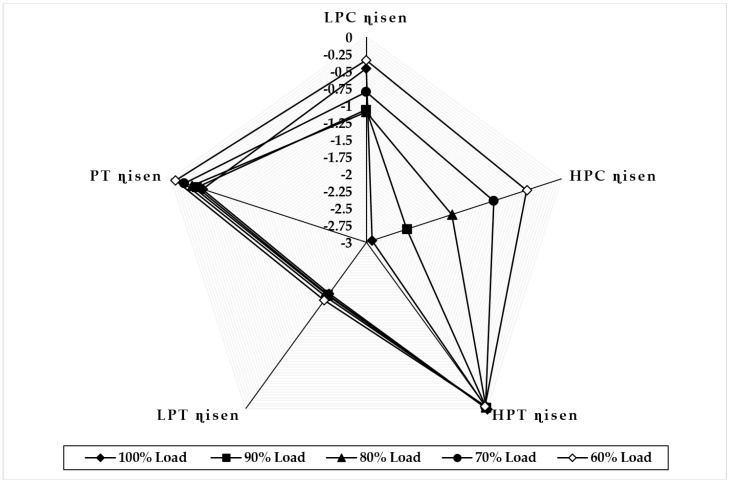
Deviation in component isentropic efficiency due to LPT erosion at 100% fault severity at full−load and part−load operation.

Figure 13 illustrates the variation in component isentropic efficiency when fouling at 100% severity level occurs in the LPT at full-load and part-load operations. The graph depicts that the maximum deviation in isentropic efficiency at 90% load in LPC is about 2.49%, but, at 80%, 70%, and 60% load, the maximum deviation in isentropic efficiency is occurred in LPT, about −2.50%, 2.53%, and 2.54%, respectively. The minimum deviation in isentropic efficiency shown in PT is about 0.024 at 90% load, −0.031 at 80% load, −0.072 at 70% load, and −0.051 at 60% load. The trend of the deviation in the PT isentropic efficiency is decreasing as load decreases. Figure 14 illustrates the variation in component isentropic efficiency when erosion at 100% severity level occurs in the LPT at full-load and part-load operations. The graph depicts that the maximum deviation in isentropic efficiency at 90% load is occurred in HPC, about −2.37%, and, at 80%, 70%, and 60% load the maximum deviation in isentropic efficiency is occurred in LPT is about −2.04%, 2.01%, and 1.95%, respectively. The minimum deviation in isentropic efficiency shown in HPT is about −0.008 at 90% load, −0.026 at 80% load, −0.04 at 70% load, and −0.048 at 60% load. The trend of the deviation in the HPT isentropic efficiency is decreasing as load decreases.

**Figure 15:**
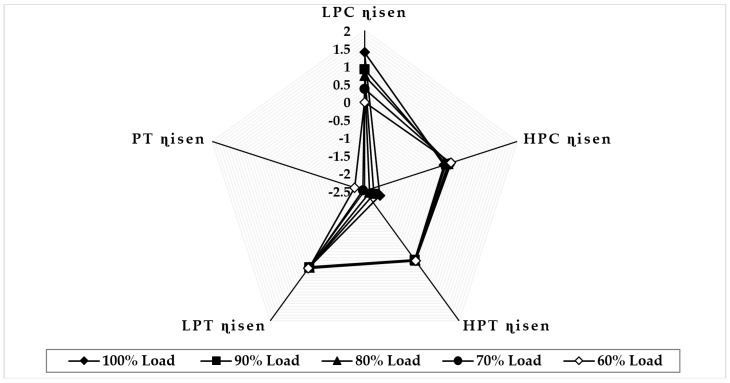
Deviation in component isentropic efficiency due to PT fouling at 100% fault severity at full−load and part−load operation.

**Figure 16:**
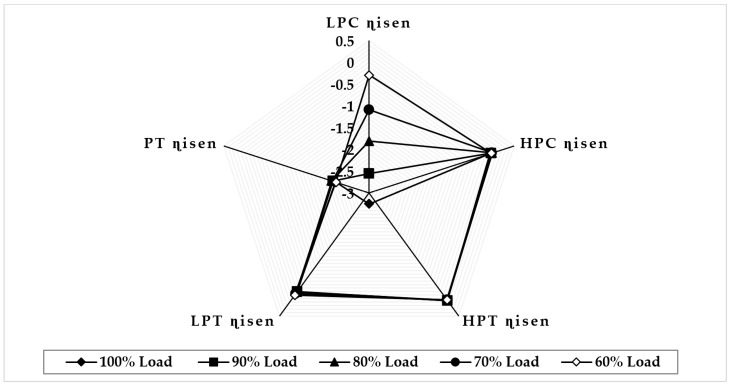
Deviation in component isentropic efficiency due to PT erosion at 100% fault severity at full−load and part−load operation.

Figure 15 illustrates the variation in component isentropic efficiency when fouling at 100% severity level occurs in the PT at full-load and part-load operations. The graph depicts that the maximum decrease in isentropic efficiency that occurs on itself is about −2.77 at 90% load, −2.64 at 80% load, −2.45 at 70% load, and −2.20 at 60% load, whereas the minimum decrease in isentropic efficiency observed in HPC isentropic efficiency is about −0.099 at 90% load, −0.031 at 80% load, 0.014 at 70% load, and 0.045 at 60% load. The trend of the deviation in PT isentropic efficiency is decreasing as load decreases, whereas HPC isentropic efficiency deviation increases as load decreases. Figure 16 illustrates the variation in component isentropic efficiency when erosion at 100% severity level occurs in the PT at full-load and part-load operations. The graph depicts that the maximum decrease in isentropic efficiency that occurs on LPC is about −2.56% at 90% load, respectively, and on PT is about −2.09 at 80% load, −2.12 at 70% load, and −2.19 at 60% load, whereas the minimum decrease in isentropic efficiency observed in HPC isentropic efficiency is about −0.085 at 100% load, −0.06 at 90% load, −0.028 at 80% load, −0.029 at 70% load, and −0.05 at 60% load. The trend of the deviation in HPC isentropic efficiency increases as load decreases. A small number of measurable gas turbine parameters are also available to monitor the gas turbine. Mohd et al. [1] recommended the top ten three-shaft gas turbine diagnosis set parameters following the correlation analysis. The following are the list: PT4, T24, P3, T3, P43, P47, T5, FF, N1, and N2. These parameters assist in determining the health status of the gas turbine component.

**Figure 17:**
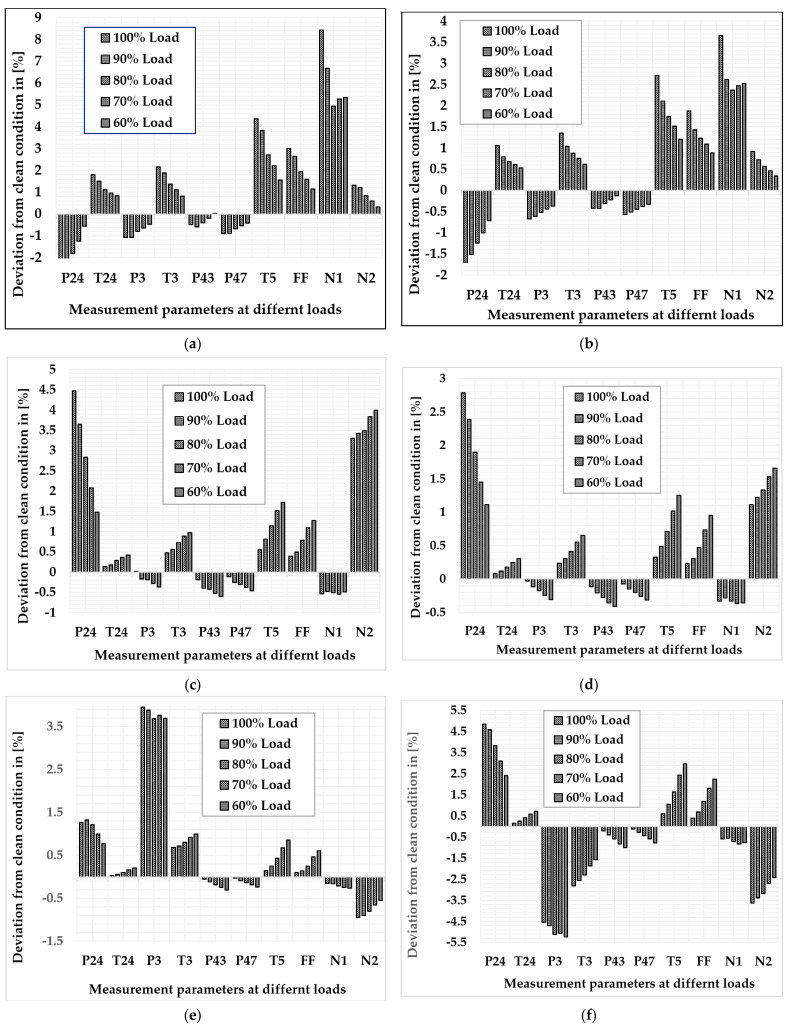

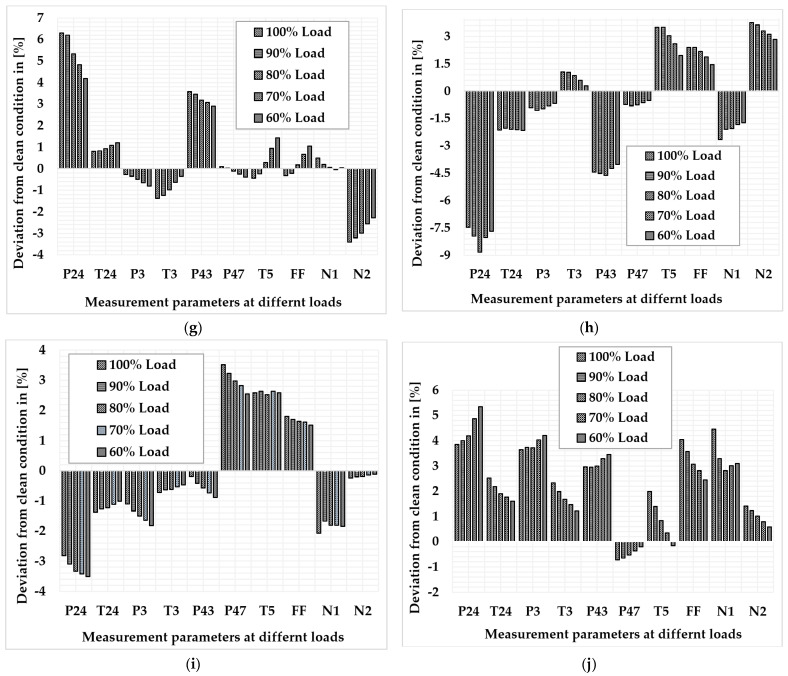
Deviation in measurement parameters due to physical faults at full-load and part-load operation: (**a**) LPC fouling at 100% fault severity, (**b**) LPC erosion at 100% fault severity, (**c**) HPC fouling at 100% fault severity, (**d**) HPC erosion at 100% fault severity, (**e**) HPT fouling at 100% fault severity, (**f**) HPT erosion at 100% fault severity, (**g**) LPT fouling at 100% fault severity, (**h**) LPT erosion at 100% fault severity, (**i**) PT fouling at 100% fault severity, (**j**) PT erosion at 100% fault severity.

Figure 17a illustrates the variation in measurement parameters when fouling occurs in the LPC at 100% fault severity. The graph revealed that the maximum deviation that occurs in low-pressure spool speed is about 6.68% at 90% load, 4.95% at 80% load, 5.27% at 70% load, and 5.33% at 60% load, whereas the minimum deviation shown in the HPT exit pressure is about −0.59% at 90% load, −0.39% at 80% load, −0.20% at 70% load, and 0.026% at 60% load. The trend of the deviation in low-pressure spool speed is fluctuating as the load decreases, but the HPT exit pressure decreases as load decreases. Figure 17b illustrates the variation in measurement parameters when erosion occurs in the LPC at 100% fault severity. The graph revealed that the maximum deviation that occurs in low-pressure spool speed is about 2.61% at 90% load, 2.36% at 80% load, 2.47% at 70% load, and 2.52% at 60% load, whereas the minimum deviation displayed in HPT exit pressure is about −0.42% at 100% load, −0.42% at 90% load, −0.31% at 80% load, −0.22% at 70% load, and −0.13% at 60% load. The trend of the deviation in low-pressure spool speed is fluctuating as the load decreases, but the HPT exit pressure continues to decrease as load decreases.

Figure 17c illustrates the variation in measurement parameters when fouling occurs in the HPC at 100% fault severity. The graph revealed that the maximum deviation at 100% load shown in the LPC exit pressure is about 3.64% at 90% load, 2.83% at 80% load, 2.07% at 70% load, and 1.47% at 60% load, whereas the minimum deviation that occurs in HPT exit pressure is about −0.26% at 90% load, −0.30% at 80% load, −0.38% at 70% load, and −0.46% at 60% load. The trend of the deviation in LPC exit pressure decreases as load decreases, whereas the deviation in the HPT exit pressure increases as the load decreases. Figure 17d illustrates the variation in measurement parameters when erosion occurs in the HPC at 100% fault severity. The graph revealed that the maximum deviation at 100% load shown in the LPC exit pressure is about 2.38% at 90% load, 1.89% at 80% load, 1.45% at 70% load, and 1.11% at 60% load, whereas the minimum deviation that occurs in HPT exit pressure is about −0.15% at 90% load, −0.20% at 80% load, −0.26% at 70% load, and −0.31% at 60% load. The trend of the deviation in LPC exit pressure decreases as load decreases, whereas the deviation in the HPT exit pressure increases as the load decreases.

Figure 17e illustrates the variation in measurement parameters when fouling occurs in the HPT at 100% fault severity. The graph revealed that the maximum deviation that occurs with HPC exit pressure is about 3.88% at 90% load, 3.68% at 80% load, 3.76% at 70% load, and 3.69% at 60% load, whereas the minimum deviation at 100% load shown in the HPT exit pressure is about −0.030%, and, at 90% load, 80% load, 70% load, and 60% load in the LPC exit temperature, the values are about 0.06%, 0.10%, 0.166%, and 0.21%, respectively. The trend of the deviation in the HPC exit pressure is fluctuating as the load decreases, but the LPC exit temperature increases as load decreases. Figure 17f illustrates the variation in measurement parameters when erosion occurs in the HPT at 100% fault severity. The graph revealed that the maximum deviation at 100% load shown in the LPC exit pressure is about 4.6% at 90% load, 3.83% at 80% load, 3.1% at 70% load, and 2.42% at 60% load, whereas the minimum deviation at 90% load, at 80% load, at 70% load, and at 60% load shown in the LPC exit temperature is about 0.26%, 0.41%, 0.59%, and 0.73%, respectively. The trend of the deviation in LPC exit pressure decreases as load decreases.

Figure 17g illustrates the variation in measurement parameters when fouling occurs in the LPT at 100% fault severity. The graph revealed that the maximum deviation that occurs in LPC exit pressure is about 6.20% at 90% load, 5.32% at 80% load, 4.82% at 70% load, and 4.18% at 60% load, whereas the minimum deviation at 90% load shown in the LPT exit pressure is about 0.018%, and, at 80%, 70%, and 60%, the load shown in the low-pressure spool speed is about 0.07%, −0.036%, and 0.041%, respectively. The trend of the deviation in LPC exit pressure shows decrease as load decrease, whereas the trend of low-pressure spool speed fluctuates as the load decreases. Figure 17h illustrates the variation in measurement parameters when erosion occurs in the LPT at 100% fault severity. The graph revealed that the maximum deviation that occurs in LPC exit pressure is about −7.94% at 90% load, −8.82% at 80% load, −8.02% at 70% load, and −7.68% at 60% load, whereas the minimum deviation at 100%, 90%, and 80% load shown in the LPT exit pressure, about −0.72%, −0.81%, and −0.76, respectively, but, at 70% and 60% load, the minimum deviation shown in the HPC exit temperature, about 0.58% and 0.3%, respectively. The trend of the deviation in LPC exit pressure fluctuates as load decreases, whereas the trend of HPC exit temperature decreases as the load decreases.

Figure 17i illustrates the variation in measurement parameters when fouling occurs in the PT at 100% fault severity. The graph revealed that the maximum deviation at 100% and 90% load shown in the LPT exit pressure is about −3.5%, 3.23%, and −0.76, respectively, at 80%, 70%, and 60% load, the maximum deviation shown in the LPC exit pressure is about −3.33%, −3.41%, and −3.51%, respectively, whereas the minimum deviation at 90%, 80%, 70%, and 60% load shown in the high-pressure spool speed is about −0.21%, −0.19%, −0.14%, and −0.115%, respectively. The trend of the deviation in LPC exit pressure increases as the load decreases, whereas the trend of high-pressure spool speed decreases as the load decreases. Figure 17j illustrates the variation in measurement parameters when erosion occurs in the PT at 100% fault severity. The graph revealed that the maximum deviation at 90%, 80%, 70%, and 60% load shown in the LPC exit pressure is about 3.99%, 4.18%, 4.86%, and 5.34%, respectively, whereas the minimum deviation at 90%, 80%, and 70% load shown in the LPT exit pressure is about −0.64%, −0.53%, and −0.37%, respectively, but, at 60% load, the minimum deviation shown in the PT exit temperature is about −0.15%. The trend of the deviation in LPC exit pressure increases as the load decreases, while the trend of LPT exit pressure decreases as the load decreases.

**Figure 18:**
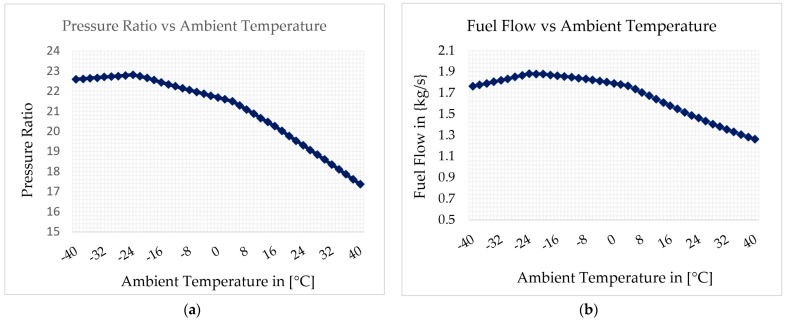
The effect of ambient temperature variation: (**a**) on pressure ratio, (**b**) on fuel flow.

Finally, the effect of ambient temperature on two key gas turbine parameters, pressure ratio and fuel flow, was simulated in the off-design model. Figure 18a,b below shows the results.

Pressure ratio versus ambient temperature and fuel flow versus ambient temperature are shown in Figure 18a and Figure 18b, respectively. The simulation result shows that both pressure ratio and fuel flow decrease when ambient temperature increases. However, exceptionally, both pressure ratio and fuel flow increase when ambient temperature varies from −40 °C to −24 °C. It is worth noting that this parametric simulation is conducted with the optimized off-design model, which provides accurate results in different scenarios or at all operating points.

## 4. Conclusions

The results demonstrated that the low-pressure compressor’s condition of health is one of the most important factors. Its decline has an enormous effect on other components’ health status. Additionally, it was found that fouling has a greater impact on the upstream components, while erosion has a greater impact on the downstream components. It was also proved that the downstream components are more significantly impacted by the upstream components. Furthermore, the results of the simulation demonstrated that the variable inlet guide vane significantly helps to improve the gas turbine’s performance, particularly the performance of the low-pressure compressor and low-pressure turbine, because the variable inlet guide vane is connected to the low-pressure compressor and the low-pressure turbine is connected to the low-pressure compressor via a common shaft. With normalized speed, the variable inlet guide vane is programmed. When the normalized speed lowers because of fouling or erosion, the variable inlet guide vane can be opened more to increase the air mass flow rate and maintain efficiency. The best diagnosis set parameters were observed. The simulation results show that fuel flow rate, high- and low-pressure spool speed, LPC and HPC exit pressure, and PT exhaust temperature show more deviation than other parameters for both faults. The full-load and part-load simulation results show that the deviation in some measurement parameters from the clean condition is nonlinear. It differs from component to component and from physical fault to physical fault, which is clearly shown in the results. The results are useful to improve understanding regarding gas turbine performance deterioration at full-load and part-load operation, and they are also helpful in the investigation of the root cause of performance degradation, which is highly significant during development of a fault detection and isolation model. The authors recommend investigating the combined effect of physical faults on the gas turbine performance at full-load and part-load operation in future research.

## Figures and Tables

**Table 1:** Input data for design point model.

Parameter	Unit	Value	Source
Power output	MW	26.025	OEM document
Pressure ratio	-	20:01	OEM document
Thermal efficiency	%	35.8	OEM document
Exhaust mass flowrate	Kg/s	92.2	OEM document
Heat rate	KJ/KWh	10043	OEM document
Turbine inlet temp.	°C	1193	[1]
Exhaust temperature	°C	488	OEM document
LPC rotational speed	RPM	6643	OEM document
HPC rotational speed	RPM	9445	OEM document
FPT rotational speed	RPM	4950	OEM document
LPC stages	-	7	OEM document
HPC stages	-	6	OEM document
HPT stages	-	1	OEM document
LPT stages	-	1	OEM document
FPT stages	-	2	OEM document

**Table 2:** Parameters used as constraints.

Constraints	Min Value	Optimized Values	Max Value
Overall thermal efficiency (%)	33	35.85	41
Heat rate (KJ/KWh)	8853	10,040.2	10,043.5
Exhaust Temperature (°C)	460	479.076	496.7

**Table 3:** Parameters used for optimization.

Variables	Minimum Value	Optimized Value	Maximum Value
HPT NGV1 Cooling air	0.04	0.061	0.065
HPT Rotor 1 Cooling air	0.03	0.051	0.054
IPT NGV 1 Cooling air	0.008	0.021	0.025
IPT NGV 1 Cooling air	0.008	0.021	0.025
Exhaust pressure ratio	1	1.1620	1.2
IPC Isentropic Efficiency	0.9	0.9	0.95
HPC Isentropic Efficiency	0.9	0.85	0.95
HPT Isentropic Efficiency	0.89	0.8977	0.93
LPT Isentropic Efficiency	0.91	0.9125	0.94
PT Isentropic Efficiency	0.89	0.8963	0.92

**Table 4:** Objective function.

Parameter	Value
Power output (KW)	26,025

**Table 5:** Comparison of the design point model output with OEM catalogue data.

Parameter	Units	OEM Data	GasTurb13 Model	% Error
Power Output	kW	26,025	26,025.5	0.0019
Thermal Efficiency	%	35.8	35.8	0
Pressure Ratio	-	20:1	20:1	0
Fuel Flowrate Lower Heating Value	kg/s MJ/kg	- -	1.53281 47.16	- -
Exhaust Temperature	°C	488	479.5	1.116
Turbine Inlet Temperature	°C	1193	1193	0
Heat Rate	kJ/(kWh)	10,043	10,040.2	0.027

**Table 6:** Spool speeds.

	Low-Pressure Spool	Intermediate Pressure Spool	High-Pressure Spool
Absolute [RPM]	4950.0	6643.0	9445.0
Relative	1.0000	1.0000	1.0000

**Table 7:** Surge margin [%].

Low-Pressure Compressor Map	Low-Pressure Compressor Map
56.467	24.104

**Table 8:** Map coordinates.

	LPC	HPC	HPT	IPT	PT
Map relative Speed	1.0000	1.0000	1.0000	1.0000	1.0000
Map Coordinate Beta	0.4000	0.5000	0.5000	0.5000	0.7000

**Table 9:** Relationship between the physical fault and health parameters.

Physical Fault	Flow Capacity Change (A)	Isentropic Efficiency Change (B)	Ratio A:B	Range
Compressor fouling	Γ_C_↓	η_C_↓	3:1	(0, −7.5%) (0, −2.5%)
Compressor erosion	Γ_C_↓	η_C_↓	2:1	(0, −4%) (0, −2%)
Turbine fouling	Γ_T_↓	η_T_↓	2:1	(0, −4%) (0, −2%)
Turbine erosion	Γ_T_↓	η_T_↓	2:1	(0, +4%) (0, −2%)

## Data Availability

Data available on request due to restrictions.

## Nomenclature

CC	Combustion chamber
GT	Gas turbine
FF	Fuel flow
HPT	High-pressure turbine
HPC	High-pressure compressor
ISO	International Standard Organization
LPC	Low-pressure compressor
LPT	Low-pressure turbine
N1	Low-pressure speed
N2	High-pressure speed
NGV	Nozzle guide vane
OEM	Original equipment manufacturer
PT	Power turbine
P24	Exit pressure of low-pressure compressor
P3	Exit pressure of high-pressure compressor
P43	Exit pressure of high-pressure turbine
P47	Exit pressure of low-pressure turbine
T24	Exit temperature of low-pressure compressor
T3	Exit temperature of high-pressure compressor
T5	Exit pressure of power turbine
VIGV	Variable inlet guide vane
